# Opportunistic genomic screening. Recommendations of the European Society of Human Genetics

**DOI:** 10.1038/s41431-020-00758-w

**Published:** 2020-11-22

**Authors:** Guido de Wert, Wybo Dondorp, Angus Clarke, Elisabeth M. C. Dequeker, Christophe Cordier, Zandra Deans, Carla G. van El, Florence Fellmann, Ros Hastings, Sabine Hentze, Heidi Howard, Milan Macek, Alvaro Mendes, Chris Patch, Emmanuelle Rial-Sebbag, Vigdis Stefansdottir, Martina C. Cornel, Francesca Forzano, On behalf of the European Society of Human Genetics

**Affiliations:** 1 Department of Health, Ethics and Society, CAPHRI Care and Public Health Research Institute, and Research School GROW for Oncology & Developmental Biology, Maastricht University, Maastricht, The Netherlands; 2 Institute of Medical Genetics, Division of Cancer & Genetics, School of Medicine, Cardiff University, Cardiff, UK; 3 Biomedical Quality Assurance Research Unit, Department of Public Health and Primary Care, University of Leuven, Leuven, Belgium; 4 Département de génétique, SYNLAB, Chemin d’Entre-Bois 21, 1018 Lausanne, Switzerland; 5 UK National External Quality Assessment Service for Molecular Genetics/Genomics Quality Assessment, Department of Laboratory Medicine, Royal Infirmary of Edinburgh, Edinburgh, UK; 6 Section Community Genetics, Department of Clinical Genetics and Amsterdam Public Health research institute, Amsterdam UMC, Vrije Universiteit Amsterdam, Amsterdam, The Netherlands; 7 The ColLaboratory, University of Lausanne, Lausanne, Switzerland; 8 CEQAS/GenQA, John Radcliffe Hospital, Oxford University Hospitals NHS Foundation Trust, Oxford, UK; 9 Praxis für Humangenetik, Mannheim, Germany; 10 Medical Ethics, Lund Universitet, Lund, SE-221 00 Sweden; 11 Division of Industrial Biotechnology, Department of Biology and Biological Engineering, Chalmers University of Technology, Gothenburg, 412 96 Sweden; 12 Department of Biology and Medical Genetics, Charles University and Motol University Hospital, Prague, Czech Republic; 13 UnIGENe and CGPP-Centre for Predictive and Preventive Genetics, IBMC-Institute for Molecular and Cell Biology, i3S-Instituto de Investigação e Inovação em Saúde, Universidade do Porto, Porto, Portugal; 14 Genomics England, Queen Mary University of London, London, UK; 15 Society and Ethics Research Group, Connecting Science, Wellcome Genome Campus, Cambridge, CB10 1SA UK; 16 Laboratoire d‘Épidémiologie et de Santé Publique, UMR 1027 INSERM, Université Paul-Sabatier, Toulouse, France; 17 Department of Genetics and Molecular Medicine, Landspitali University Hospital, Reykjavik, Iceland; 18 Clinical Genetics Department, Guy’s & St Thomas’ NHS Foundation Trust, London, UK

**Keywords:** Medical ethics, Genetics

## Abstract

If genome sequencing is performed in health care, in theory the opportunity arises to take a further look at the data: opportunistic genomic screening (OGS). The European Society of Human Genetics (ESHG) in 2013 recommended that genome analysis should be restricted to the original health problem at least for the time being. Other organizations have argued that ‘actionable’ genetic variants should or could be reported (including American College of Medical Genetics and Genomics, French Society of Predictive and Personalized Medicine, Genomics England). They argue that the opportunity should be used to routinely and systematically look for secondary findings—so-called opportunistic screening. From a normative perspective, the distinguishing characteristic of screening is not so much its context (whether public health or health care), but the lack of an indication for having this specific test or investigation in those to whom screening is offered. Screening entails a more precarious benefits-to-risks balance. The ESHG continues to recommend a cautious approach to opportunistic screening. Proportionality and autonomy must be guaranteed, and in collectively funded health-care systems the potential benefits must be balanced against health care expenditures. With regard to genome sequencing in pediatrics, ESHG argues that it is premature to look for later-onset conditions in children. Counseling should be offered and informed consent is and should be a central ethical norm. Depending on developing evidence on penetrance, actionability, and available resources, OGS pilots may be justified to generate data for a future, informed, comparative analysis of OGS and its main alternatives, such as cascade testing.

## Introduction

It is expected that in the near future many individuals with an indication for genetic testing will have their exome or now increasingly their entire genome sequenced (in this document referred to as: ‘genome sequencing’), also depending on the development of the total costs of diagnostic clinical sequencing. Of course, genome sequencing allows targeted bioinformatics analysis of the raw sequencing data (including uninterpreted data e.g., in Variant Call Format (VCF) files) by using “virtual panels” or ‘phenotype filters’ that are targeting genes most likely to be associated with the symptoms of an individual. During such a targeted analysis unsolicited, incidental findings may emerge, i.e., those which are unrelated to the primary clinical indication for genome sequencing. Debate is ongoing about the pros and cons of broadening the analysis by actively looking for additional variants, unrelated to the initial purpose of testing, which however could be relevant for the health prospects and/or reproductive choices of the patient or the patient’s family (so-called ‘secondary findings’; SFs). Such discussions deal with medically ‘actionable’ information associated with SFs which could help prevent a disease from occurring, or facilitate the early management of a disease once it develops (e.g., utilizing ‘precision medicine‘ approaches), diagnose a disease which is already present but has not manifested clinically, thus far, or inform reproductive decisions.

Previous recommendations issued by the European Society of Human Genetics (ESHG) on ‘Whole genome sequencing in health care’, did not explicitly explore the analysis of SFs. The ESHG document stated that within the health care context, genomic sequencing should focus on the original test indication aimed at the identification of the underlying genetic etiology of a disease and be ‘as targeted as possible’; at least for the specific clinical and technical context of genome sequencing at the time this consensus statement was published [1]. This implied not actively looking for SFs. The European stance has to be understood in the context of genome sequencing in health care, where many European countries have collectively funded health-care systems. In this regard, screening programs undergo evaluation of pros and cons before being implemented at a regional or national level depending on the organization of health care in the given European country. Screening programs tend to have limited funding from public resources, and policy decisions to embark on one new activity typically demand the balancing of health care expenditure elsewhere. In line with the ESHG recommendations, cautionary policy statements were issued at that time also by several national societies and authorities, such as the German Society of Human Genetics [2], the Health Council of the Netherlands [3], and the Canadian College of Medical Geneticists [4]. Recently the French Agency of Biomedicine document [5] is a further instance of this.

However, concurrently to the ESHG document, the American College of Medical Genetics and Genomics (ACMG) recommended a deliberate search for, and routine analysis of,  a predefined set of ‘actionable’ genomic variants in each case of exome or genome sequencing irrespective of the medical indication for such testing [6]. ACMG uses the term ‘opportunistic screening’ for this purpose, with the word ‘opportunistic’ referring to the opportunity arising with the availability of the raw genome sequencing-based data of individuals undergoing some form of genome sequencing in the context of health care for ‘secondary analyses’. In the wake of the ACMG recommendations, variations of this approach have also been proposed or implemented in different European countries, including the United Kingdom (100,000 Genomes Project, ongoing) and France [7]. These initiatives have sparked debate about the ethics of these strategies [8–10], also leading to research projects aimed at charting the ethical, legal, and social issues (ELSI) linked with opportunistic screening in genomic medicine [11–15].

Opportunistic screening should be distinguished from the use of selected multi-gene test panels in a clinical, diagnostic context. These are still currently utilized in order to decrease analytical-, bioinformatic-, and data-storage related costs and/or to increase specific target sequence coverage and thus the analytical robustness of genetic testing. The following example illustrates this distinction: If the indication for sequencing involves an oncological problem which could be part of a specific rare hereditary cancer syndrome, the applied test panel comprises multiple disease genes associated with such syndromes, thereby reflecting clinical/laboratory and genetic knowledge at a specific time point. However, such broader scale genetic analysis still remains within the frame of the diagnostic purpose of such testing. By contrast, testing for ‘cancer predispositions’ not linked with the suspected tumor syndrome(s) in question would amount to ‘opportunistic screening’.

The ESHG regards it as its professional responsibility to contribute to this ongoing debate. The present document specifically discusses the pros and cons of opportunistic genomic screening (OGS), understood as the deliberate search for genetic variants unrelated to the diagnostic question. The wider discussion of dealing with unsolicited findings (UFs) in genomic medicine is beyond the scope of the analysis presented here. This new ESHG position statement contains relevant background information, ethical reflection, and updated recommendations. A working group of the ESHG’s Public and Professional Policy Committee (PPPC) prepared the draft, which was then discussed by PPPC and experts from the ESHG-EuroGentest Committee and Quality subcommittee (https://www.eshg.org/index.php?id=55). It was sent to ESHG members and selected experts to solicit comments from 20 April until 20 May 2020. The authors have subsequently integrated the suggestions where appropriate. The Board of ESHG has approved the final version on 19 July 2020. In view of rapid developments in the field and given the need for further reflection, these Recommendations will need regular evaluation in the future.

## Opportunistic screening in genomic medicine

### ‘Opportunistic screening’ and ‘secondary findings’

The concept of opportunistic screening is not new. For instance, in Family Medicine, general practitioners make use of patient-initiated consultations to test routinely for e.g., high blood pressure or analyze serum glucose/cholesterol concentrations when screening for the metabolic syndrome. The concept of opportunistic screening has also been used in radiology, e.g., when assessing the degree of osteoporosis during computed tomography scans for other indications [16]. When such tests are performed in patients without a clinical indication for such testing, this amounts to a form of screening. What makes it ‘opportunistic’, is that those who might benefit from testing are only those who happen to contact medical services for whatever reason. Opportunistic screening differs from programmatic screening, where all members of a predefined target population are systematically invited for a uniformly organized and externally evaluated screening service.

For the tested individuals, opportunistic screening does not necessarily entail undergoing medical/laboratory procedures that they would otherwise not be subjected to. It may imply carrying out an extra test (e.g., determining the blood pressure) or extra venepuncture (e.g., examine serum glucose/cholesterol concentrations). It may also consist of an extended analysis of the data resulting from indicated testing, as for instance when a doctor instructs the laboratory to check for a wider range of disease markers in a blood test than those needed in view of a specific medical indication for which the test was ordered. Opportunistic screening as discussed in this document, is of the latter kind: it involves a wider analysis of the raw sequencing data that are available when clinical genome sequencing is being performed.

In genomic medicine, opportunistic screening consists of a routine search for SFs, so called to mark the difference from those answering (or partly answering) the clinical question (‘primary findings’). Conceptually, SFs are also to be distinguished from ‘incidental findings’ (IFs). Although both terms (SF and IF) refer to results unrelated to the original reason for testing, SFs are actively sought for, whereas IFs are not. In the context of Next Generation Sequencing (NGS), IFs are not necessarily rare, and the ESHG has suggested that ‘UFs’ is a more appropriate descriptive term than IFs [1]. In this document we use the term ‘OGS’ to refer to the active or deliberate search for SFs in the context of genome sequencing in health care.

### Selected OGS-proposals and practices

This section summarizes three examples of OGS proposals and practices, starting with the relevant recommendations of the ACMG, as these may be considered as an initial frame of reference. Two further examples of OGS are drawn from France and the United Kingdom.

#### ACMG recommendations

The ACMG proposal recommends that laboratories performing genome sequencing seek and report to the physician a minimum list of highly penetrant, actionable variants in preselected candidate genes, regardless of the indication for which the clinical sequencing was ordered and irrespective of the age of the patient [6]. Although the relevant ACMG Working Group recommended reporting only variants with a high likelihood of causing disease, it recognized at the time “that there are limited data available in many cases to make this assessment”, i.e., there was little information on respective variant penetrance and/or expressivity. While the “original” minimum list originally entailed 57 clinically relevant genes, this list was later decreased to 56 and then subsequently expanded to 59 [17]. The specific genes under consideration pertain broadly to two major medical domains, i.e., genes predisposing to specific forms of cancer and those predisposing to cardiac diseases where presymptomatic medical interventions may be of relevance. The ACMG recommends refining and updating this list at least annually, based on developing scientific and medical evidence. Depending upon the specific genetic risk factor or variant, carriers can make use of individualized preventive options, including early or long-term medical imaging-based monitoring, colonoscopy, prophylactic surgery, and utilization of implantable cardioverter-defibrillators. After some debate about the extent to which patients should be given a choice, ACMG currently advocates an ‘opt out’ approach for patients who only want information relevant to the original indication (i.e., ‘purpose’) for genome sequencing [17]. Given the ACMG’s assessment of large benefits and minimal risks, it would, as stated in a later clarification document, be unethical not to offer OGS [18]. In a supporting paper it is said that the ACMG Recommendations may count “as evidence of the standard of care” in the case of malpractice litigation [19].

#### The French Society of Predictive and Personalized Medicine (SFMPP) recommendations

The SFMPP published its “Guidelines for reporting SFs of genome sequencing in cancer genes” in August 2018 [7]. It discusses multi-gene panels aimed at familial tumor syndromes, including genes unrelated to the patient’s tumor. The document speaks of SFs as “the results of a deliberate (…) screening for alterations in genes that are not relevant to the diagnostic indication for which the screening was ordered.” As a consequence, the guidelines fit in with the concept of OGS, as defined above. Using the criterion of ‘actionability’, an evaluation of the relevant risk and the level of evidence, the SFMPP provisionally recommends reporting information on 36 (so-called ‘class 1’) genes related to specific forms of cancer in adults. While there is significant overlap with the ‘cancer genes’ on the ACMG list, the SFMPP lists many additional genes, for instance *PALB2*, while excluding all the ‘cardiac genes’. With regard to patient autonomy, the SFMPP insists on an explicit informed consent procedure, rather than a mere opt-out procedure. The document recommends a system of multi-step (‘dynamic’) consent. The first step is in the context of pretest counseling where patients are asked to indicate whether they want to be informed about SFs in this subset of genes or not. The second step is when patients are being informed about the primary results. Here, they are given the opportunity after further reflection (“with more autonomy”) to confirm or refuse access to the information resulting from the search for SFs. This two-step counseling approach was proposed by patient associations in order to limit the potential psychological impact of OGS. The SFMPP recommendations are limited to OGS in adults, pending further debate and reflection on the acceptability of OGS for cancer-related genes in minors.

We present here the SFMPP guidelines as an illustration of a further OGS-proposal, while being aware that in France, as elsewhere in Europe, the debate about the pros and cons of OGS is still going on. Thus, the French Agency of Biomedicine has recently adopted a draft of recommendations for good practice with respect to the additional data generated by NGS [5], which awaits ratification by the French Ministry of Health, and state that “At the present state of scientific knowledge, it is recommended not to propose, in a diagnostic setting, a systematic analysis of genes that are not related to the initial indication based on a pre-established list”.

#### 100,000 Genomes project and National Health Service (NHS) England Genomic Medicine Service

The 100,000 Genomes Project (100 KGP) was initiated in 2013 with the aim of developing the implementation of DNA sequencing technologies and thereby embedding genomic medicine into routine health care. Recruitment into 100 KGP was primarily of patients with undiagnosed rare disease or with specific cancers and this was completed in 2018. The NHS England Genomic Medicine Service is being instigated, building on learning from the 100 KGP, and introducing whole genome sequencing as a clinical test in the NHS in England [20]. In October 2018, the UK Secretary of State for Health and Social Care stated an ambition to achieve the sequencing of 1 million genomes by the NHS and the research project UK Biobank over 5 years, including those with rare diseases and cancers, including a population cohort [21].

Participants in 100 KGP gave consent for genome sequencing with return of results related to their presenting condition and the use of their data for research. Those recruited were also offered the return of a limited set of additional ‘looked for’ findings which would be confirmed by accredited clinical diagnostic laboratories and then usually returned to the patients by the specialist who had recruited them to the 100 KGP. These SFs would be generated via a separate bioinformatics analysis on the genomes of all those who had consented. There were two classes of SFs: (i) medically actionable information (such as Lynch syndrome) and (ii) information of reproductive significance (in particular Cystic Fibrosis carrier status). Participants could make the same or different decisions about the two categories of SFs and they could also change their minds at any time. Consent is sought for findings that were described as actionable rather than specific, named conditions and participants were informed that any conditions tested for would be serious and that prevention or treatment was available in the NHS. The offer to participants in relation to reproductive findings (i.e., carrier status) was framed as looking for variants that would not affect the individual but could affect their future children [22].

In addition to questions of ‘actionability’, other factors were considered in drawing up the list of SFs. Only those disease genes were included which comprise high penetrance variants and where the association with disease and/or the evidence for the efficacy of interventions was strongly substantiated; where it would be technically possible to reliably detect variants in these genes using genome sequencing, variants would only be reported where there was a high confidence that they would be pathogenic or likely pathogenic (capable of affecting function and causing disease in a specific context). In addition, evidence of clinical benefit from application of the genomic information would be required, not simply the validity of the information itself. This takes account of the potential burden on NHS staff in validating and returning findings, and whether care pathways for patients are established within the NHS. However, the scenario of OGS crowding out resources for indication-based pathways remains a matter of concern in collectively funded health-care systems, such as in the UK.

In line with current recommendations on genetic testing in children, the search for additional health-related findings in minors is restricted to conditions where benefit could be assumed during childhood and carrier testing is not offered.

At the time of writing (May 2020) the return of additional findings from 100 KGP has been delayed, but is expected to go ahead in 2020. The return of SFs will be accompanied by research on the feasibility and acceptance of this procedure, together with a health economics assessment. However, a decision regarding whether a similar process of returning additional ‘looked for’ findings will be offered in the NHS Genomic Medicine Service has not yet been made.

## Ethical exploration

In view of an ethical evaluation of OGS as exemplified in the above proposals or practices, a preliminary question is how they should be conceptualized for normative purposes. There is more than one way of doing so, depending on which elements are regarded as normatively relevant.

Firstly, the fact that OGS is carried out in the context of individual patient medical care makes it a kind of in-between concept: ‘screening’ in so far as the active search for SFs goes beyond the original indication for testing, and ‘individual care’ in so far as this search is aimed at enhancing the medical benefits of a clinical test for the patient. The ACMG strongly emphasizes the latter perspective [6]. It stresses that its recommendations ought to be regarded as part of medical doctors’ fiduciary duty, i.e., as a matter of providing good clinical care to the patient, who would naturally expect the doctor to actively look for (actionable) information relevant to his or her health. To the extent that this does amount to screening, this is seen as different from the kind of screening to which the normative framework applies that was developed by the WHO (‘Wilson and Jungner’) [23] and other national and international authorities [24]. The difference being precisely that this framework was meant for organized screening programs targeting population groups in a public health context [25], rather than for the clinical context for which OGS is being proposed [26]. However, this may be too swift a dismissal of the wider relevance of this framework also for OGS. From a normative perspective, the distinguishing characteristic of medical screening is not so much the context in which it is performed (whether public health or health care), but the lack of an indication for having this specific test or investigation in those to whom screening is offered [27]. As famously stated by Cochrane and Holland [28], the non-indicated nature of screening entails a more precarious benefits-to-risks balance in comparison to indication-based testing: “If a patient asks a medical practitioner for help, the doctor does the best he can. He is not responsible for defects in medical knowledge. If, however, the practitioner initiates screening procedures he is in a very different situation. He should in our view, have conclusive evidence that screening can alter the natural history of disease in a significant proportion of those screened”. In view of this difference, the core requirements of the traditional screening framework include (1) evidence that for those being screened, this balance is clearly favorable (proportionality) and (2) explicit informed consent by those to whom the screening offer is made (autonomy). Moreover, especially when screening is offered in the context of collectively funded health care it requires (3) a justification in terms of considerations of distributive justice.

Secondly, given that what we are dealing with here is the wider analysis of raw sequencing data that have become available as a result of testing, a further possible understanding is that providing this information is a matter of the individuals’ right to information that others have obtained about them. However, this seems to ignore the difference between raw sequencing data and whatever meaningful genomic information can be extracted from those data, either with clinical or personal utility. Even if the patient has a right to his or her raw data (including e.g., VCF files), it does not follow that medical professionals should perform the analysis needed to turn that data into information. If they decide to do so, this requires a separate justification, which leads back to the above discussion of OGS as a form of screening in the context of clinical care.

We intend to contribute to further debate about the conditions for responsible OGS by considering how such an offer relates to the three core requirements of the screening framework: proportionality, autonomy, and justice, while differentiating between OGS as offered to adults and as offered to children (or minors) [12].

### OGS offer to competent adult patients

#### Proportionality

Since OGS is offered to those who do not have a medical problem or medical history-based reason for having the relevant sequencing data analyzed, and because generating medical information may also have adverse effects, it is not obvious that a specific OGS proposal is on balance beneficial for those to whom the offer to search for SFs is made. Whether it is, can only be determined based on scientific evidence—not just considering the potential benefits that it may yield, but also specifying the possible harms that it may bring. Given that OGS is a form of genetic screening, any benefits and harms may affect not just the individual whose genome data are analyzed, but their genetic relatives as well. Notwithstanding the requirement that the proportionality balance must be positive for the persons being screened in the first place, these ‘third party’ effects should be considered as well.

##### Possible benefits

The possible benefits of OGS are primarily medical. First and foremost, OGS is aimed at yielding information allowing the primary or secondary prevention of serious genetic diseases where early or presymptomatic medical interventions could prevent their development or at least substantially delay their onset, notably forms of hereditary cancer syndromes and cardiogenetic disorders, not only in the screened individual with a ‘positive’ result, but also their genetic relatives. A recent study provided evidence that 2.6% of healthy individuals would be shown to carry an increased risk for a severe dominant disease if routinely screened for variants in the ACMG minimum list of genes [29]. The health benefits following from this may be considerable, depending, however, on several factors. The positive predictive value of the SFs targeted in the OGS panel must be high, the effectiveness of the preventative interventions or measures recommended to those found to be at risk should be scientifically proven, and access to those interventions as well as to relevant counseling must be guaranteed. Whether the latter conditions will be appropriately met, is contextually dependent on the health-care system.

A second type of medical benefit regards a more favorable risk-benefit ratio of medical interventions or treatments that the patient might have to undergo somewhere in the future. These include screening for genetic variants causing serious adverse reactions to anesthetics (already included in the ACMG list) or for pharmacogenomic (PGx) variants. As argued in one of the updated versions of the ACMG recommendations, the latter may be especially relevant where concerning “variants related to commonly prescribed medications as well as medications associated with serious adverse events for which there is greater urgency surrounding actionability” [17].

In addition to direct or future health benefits, OGS may, thirdly, provide reproductive benefits, in so far as any positive findings allow the persons being screened or their relatives to make informed reproductive choices (e.g., aimed at avoiding the conception or birth of a child with a serious genetic disorder). The current inclusion of cystic fibrosis-carrier status in the OGS approach taken in the UK represents a limited step in this direction.

A further increase of possible benefits is conceivable if more variants will be found to meet the criteria of pathogenicity and actionability and the list would be expanded. Apart from single genetic variants, future incorporation of genome-wide polygenic risk scores (PRS) might be considered if these would be shown to have clinical utility in order to reduce the risk of developing common disorders like diabetes type 2 and coronary heart disease [30]. Likewise, the reproductive benefits of OGS may be enlarged by including carrier status for a potentially large number of serious recessive disorders.

##### Risks

The potential harms and disadvantages of OGS are of different but interrelated categories: psychological, social, and medical. Some of these are of a more general nature, linked with OGS per se, while others depend on the context, content, and conditions of specific OGS-practices.

Both psychological and medical harms may arise when OGS is introduced based on insufficient evidence regarding the health impact (e.g., pathogenicity, penetrance, and expressivity) of variants in the listed disease genes. Looking for such variants in unaffected individuals has been criticized for lack of validation for general population screening; the penetrance might be lower and uncertain in the absence of family history [8, 31]. Also, ACMG underlines the importance of ongoing research into penetrance and expression (range of severity) [26]. Clearly, the penetrance of some of the variants in genes on the ACMG list has been overestimated [32–34]. Given that OGS is offered to individuals that do not have a higher a priori risk than the general population with regard to the SFs on the list, penetrance figures based on data from affected families may overestimate their risk of actually developing the disorder [35]. For instance, in 2004 the penetrance of pathogenic variants in the *SDHB* gene (succinate dehydrogenase B, causing pheochromocytoma and paraganglioma) was estimated to be 77% by 50 years of age [36], however, two recent papers concluded that in healthy relatives (“non-probands”) it is closer to 20% by 50 years of age [37, 38]. Overestimation of the health risks related to OGS findings may lead to unnecessary anxiety. It may also lead to the persons being screened being unnecessarily exposed to iatrogenic harms of invasive procedures undertaken as diagnostic or preventive measures [39], or to psychological distress of long-term surveillance. Variants have been misclassified as pathogenic on the basis of the understanding at the time of testing for both tumor syndromes and cardiogenetic conditions, especially in ethnically diverse populations [40]. This is not to deny that the penetrance of genetic variants in the general population (although it might be lower than their penetrance in affected families) may still be sufficiently high to warrant their inclusion in OGS. If so, the lower magnitude of risk may well require preventive strategies that reflect a different proportionality balance as compared to prevention in affected families.

In order to avoid that people being screened are confronted with the psychological burden of being told they are at risk of developing a serious disorder for which no options for treatment or prevention exist, OGS proposals rightly insist on the condition that to qualify for OGS, SFs should also be ‘actionable’. However, psychosocial harm may still ensue when actionability is too easily assumed [9], or when only limited actionability is taken as a sufficient reason for inclusion in the list of targeted SFs. A good example of this is the only ‘partial’ actionability of (germline) *TP53* pathogenic variants predisposing for Li-Fraumeni syndrome [41].

Assuming that OGS is only offered for SFs where there is sufficient evidence of a significant health impact (in terms of pathogenicity and penetrance) and a clear actionability (in terms of options for treatment and prevention promising to considerably ameliorate the health prospects for those with positive findings), OGS still comes with psychosocial concerns and challenges, given that little is known about how people unfamiliar with the relevant disorders will deal with positive findings and related options for prevention and reproductive choice [9]. Counseling should be provided by a professional with relevant expertise regarding the additional findings. However, we need to consider how OGS can be offered in a way that empowers people rather than undermines their confidence in their health. What are their counseling needs in connection to OGS-findings and with regard to the possible sharing of genetic information with relevant family members? Given the different setting, premature extrapolations from (mostly reassuring) psychological research in carriers in affected families should be avoided. Though some recent studies of the psychological impact of receiving ‘positive’ SFs were to some extent reassuring [42–44], more research is needed. These questions are even more important if OGS would be offered at a time when patients are trying to cope, deal with and give meaning to the totally different genetic problem for which they are having indicated clinical sequencing for example, sequencing after sudden cardiac death in a child.

The societal risks of OGS, in addition to the potential transformation of everyone who receives an SF after genetic testing into a “patient-in-waiting” [45], primarily regard possible adverse consequences for access of people ‘at high genetic risk’ to particular insurance schemes [46], or to specific jobs. Given the recent report that variants related to sudden cardiac death were found in 1% of asymptomatic individuals [47], the professions that would be at risk in this regard include bus drivers, aircraft pilots, etc. Several studies suggest that there is minimal evidence for such societal repercussions, especially when the disorders for which people prove to be at high risk are preventable or at least treatable [48]. In view of the highly different jurisdictions regarding the legal protection of applicants for jobs and insurances, these societal risks are probably to a considerable extent contextual.

As the proportionality prerequisite for offering screening is inherently linked with what has been termed the ‘evidentiary model’ [49], the ESHG observes that at least for the moment, there are simply too many questions, unknowns, uncertainties, and concerns to conclude that current OGS-proposals clearly meet this criterion—let alone that they would define the standard of care. This holds a fortiori for the suggestion to extend the concept into incorporating PRS in clinical care [30], also given the current apparent bias towards European-derived populations [50]. This is reinforced by the dependence of any putative benefits from PRS on the patient’s behavioral response to risk information, which in many studies has not been encouraging [51].

#### Respect for autonomy

After an initial debate about the extent to which a patient should be given a choice with regard to adding OGS to indicated testing [18, 52], the ACMG recommends an ‘opt out’ approach. Patients who want to have the clinically indicated sequencing without having their raw sequencing data searched for SFs, can refrain from OGS if they so wish. As this presents the search for SFs as the default position that doctors would recommend, it is at odds with the normative framework for screening, according to which the non-indicated nature of any screening offer requires those offering it to seek the full and explicit consent of those to whom the offer is made [24]. The problem with an ‘opt out’ for OGS is that patients may be insufficiently aware of the fact that the search for SFs is unrelated to the medical indication for genome sequencing, and that whether or not to have OGS is therefore something that needs separate consideration. Moreover, even when patients understand that OGS is indeed a form of screening, the message connected with offering it as a standard procedure that only some might want to opt-out from, may stand in the way of helping the patient to make a truly autonomous decision.

The SFMPP recommendations insist that “the patient’s autonomy and desire to know or to ignore SF results must be respected” and stress that the patient “could decline at any time to be informed about the SF’s even if they previously gave their approval” [7]. Pujol et al. [7] differentiate between a first step at which written consent for SF is given, a second step in which this consent is renewed (or not) and primary findings are discussed, and a third step in which the actual SFs are discussed. Such an approach may well help avoid a professional conflict of duties; it is a different matter not to screen for certain genes out of respect for the patient’s right not to know, and not to report available findings of great relevance for the patient’s own health or that of his or her close relatives [53]. Ideally, one should try to avoid burdening professionals by generating health information that the patient does not want to receive. Although this cannot be completely avoided in the genomic era, an adequate informed consent procedure for OGS should try to minimize this problem as far as possible (like in other contexts of genetic testing). For example, the raw data might only be analyzed after the second step of the SFMPP approach. This would allow tested individuals to become better informed and allow them additional time for reflection and might thereby reduce the chance that the patient later claims the right not to know about SF’s after an initial consent given for the generation of such findings, possibly generating the conflict of duties mentioned.

If genome sequencing were offered as a package of enrolment combining health care and research in a hybrid offer, where sequencing was only available if consent to research was given, then there could be concerns about so-called ‘undue inducement’, as Dheensa et al. [54] have discussed for the 100 kGP. The aims of sequencing could be blurred: both research and health care are at stake. The hybrid offer might lead people needing sequencing in a health care setting to decide to participate in sequencing because of potential advantages outside of the initial medical indication, such as receiving SFs, while also influencing them to participate in research. The hybrid nature of such initiatives raises questions concerning the consent process by distracting potential participants from its core elements and potentially violating the principle of respect for autonomy [54].

A final issue is whether patients should be given the option to decide for themselves whether to be screened for only part of the list of SFs targeted in a specific OGS offer. A categorical rejection of allowing any form of ‘personalization’ of OGS seems at odds with the principle of respect for patient autonomy. Acknowledging this principle would seem to require professionals to as much as possible respect patients’ wishes with regard to controlling what information to receive as a result of being tested. For instance, patients may want to limit the search for SFs to pharmacogenomics variants or to carrier status for recessive disorders. What patients would regard as meaningful choices in this regard and whether providing those choices would be feasible in practice is a matter for evaluation in the context of future OGS-pilots.

#### Justice

As OGS is screening in the context of health care, as it involves the further analysis of raw data that becomes available during the indicated testing, the marginal costs of screening are relatively low in comparison to establishing an entire screening program. Nonetheless, bioinformatics analysis and confirmation of detected variants (when manual variant curation and their clinical assessment will still be necessary in the foreseeable future) will remain costly despite rapid progress in machine learning-based procedures for variant prioritization. Moreover, genetic counseling costs may still be considerable given the potential need to recontact [55] and repeatedly counsel tested individuals as new evidence gradually accrues which informs the interpretation of variants [56, 57]. These aspects need to be taken into account when OGS is offered in a way that would acknowledge the principle of respect for autonomy. Costs of OGS will further increase if subsequent cascade testing among relatives of people with ‘positive’ OGS results occurs. The fact that OGS will lead to downstream costs for the health system is not problematic. However, this is a further reason for only offering OGS for variants with a proven health impact, so the costs of unnecessary interventions are avoided and the concept of ‘overdiagnosis’ as documented is dealt with e.g., in the field of radiology [58]. Moreover, in solidarity-based health-care systems, the scenario of OGS crowding out resources for indication-based care pathways raises concerns about just prioritization [59].

In view of the potential ‘add-on’ costs of OGS it is important to consider if alternative approaches could be more cost-effective. Most notably, cascade testing targeting the relatives of a proband in case of clearly pathogenic, highly penetrant, and actionable variants, such as in BRCA1- and BRCA2-related hereditary breast and ovarian cancer, Lynch syndrome and Familial Hypercholesterolaemia has been recommended. The United States Centers for Disease Control and Prevention Office of Genomics and Precision Public Health has defined such ‘Tier 1’ genomic applications as ‘having significant potential for positive impact on public health based on available evidence-based guidelines and recommendations’ [60–63]. Such cascade testing is, however, currently underutilized [64]. Whether cascade testing, OGS or a combination of both should be given priority from a distributive justice perspective is an important question that may allow for a different answer in countries that have already implemented cascade testing for a larger number of the relevant conditions in their health system, as compared to those that have not. Where OGS is used for genetic risk factors that allow for prevention through lifestyle modification rather than medical interventions, collective measures, such as general health education or measures of health protection targeting the environment or the workplace may be considered as alternatives [65]. The case for prioritizing such measures, if proven effective, is strong, especially in under-resourced countries. However, in more affluent countries, distributive justice may allow for combining collective prevention and well-defined OGS. It also needs to be noted that there remains a strong bias towards European-derived variant frequencies currently present in broadly used variant databases with data from under-resourced countries still generally missing, which may exacerbate health disparities. Individuals may potentially be harmed if there is insufficient knowledge to characterize variants as pathogenic or not in ethnically diverse populations [66].

OGS also raises a question about formal justice. OGS is only offered to individuals who happen to have an indication for genome sequencing. However, with regard to the SFs targeted in OGS these patients do not have a higher a priori risk than other members of the general population, who are not offered screening for the same conditions. This could be considered as a morally problematic inequality of access to a health service that ideally should be avoided. Along the same line it has been argued that screening for likely pathogenic and actionable variants should be offered to a general population [10]. However, as stressed by the ACMG in an earlier statement, offering the same benefits to all would come with the much higher costs of setting up the infrastructure for programmatic screening, which would be far less cost-effective than OGS [6]. Given the opportunity costs of population screening for the same set of variants, the only way of securing equality of access may well amount to denying access to all. Such ‘leveling-down’ justice (‘if not all can profit, then no one should’) is clearly not in anyone’s interest. Moreover, it could be argued that the formal justice problem of OGS is mitigated by the fact that chances of becoming a patient with an indication for clinical sequencing are equally distributed in the population. However, people who achieved higher education and income are often over-represented when applying for genetic counseling [67]. Indeed, it could be seen as exacerbating pre-existing inequities in access to health care, as described by Tudor Hart’s ‘Inverse care Law’ [68].

Further justice considerations arise with the different health care settings in which OGS could be offered. For instance, if ‘actionability’ consists of costly treatment that many people could not afford [69], screening for the relevant variants will be more beneficial for some than for others.

### OGS in minors

Traditional guidelines state that predictive genetic testing of minors should be limited to conditions where options for treatment or prevention are available that must be initiated during childhood/adolescence [70–72]. All further predictive testing should be postponed until minors are mature enough to decide for themselves about undergoing genetic testing. Arguments for this position are either based on moral rights or on morally relevant consequences. Relevant arguments of the former refer to the minor’s right to informational self-determination (as part of ‘the child’s right to an open future’). Consequence-based arguments point to how the burdens of risk status information may harm the child for example by overshadowing its psychosocial development.

To the extent that these recommendations should also apply to OGS, this significantly limits the list of variants that minors’ sequencing data could be screened for, at least where young children are concerned that have not reached sufficient maturity to decide about OGS for themselves. Although OGS for certain conditions that are actionable early in life (such as MEN type 2A, hereditary arrhythmias such as long QT and Brugada syndrome) and for pharmacogenomic variants including variants modifying the individual reaction to anesthetics could still be possible, this would rule out OGS for most of the ACMG list.

However, proponents of OGS in minors as recommended by the ACMG argue that the context for which those traditional guidelines for predictive genetic testing of children were drafted, namely presymptomatic testing in relatives of an index case, is substantially different from that of OGS [19]. In the former context, postponement of testing minors for late-onset disorders is without consequences both for them and for their relatives, as no information about their at risk status will thereby be “lost”. Refraining from OGS in minors with an indication for genome sequencing, by contrast, amounts to missing what may well be a one-off opportunity of generating potentially life-saving information both for the minors themselves and their relatives.

Whether this reasoning justifies OGS for later-onset conditions in minors is a matter for further debate. Is there evidence that minors may be harmed by telling their parents that they (i.e., those minors) are at risk of a serious but actionable later-onset disorder? If not, are the possible future benefits for the child sufficiently weighty to trump the remaining concerns about violating the minor’s right to informational privacy? How convincing in this regard is the notion of OGS as a one-off, unique, opportunity that should not be missed, given that minors will grow up in an era where genome sequencing may become a routine part of health care? Alternatively, does the argument ultimately rest on the interests that the minor’s relatives may have in not letting this opportunity for generating important health information be wasted? Then indeed the question becomes one of justifying the screening of children in order to benefit others. Are the interests of family members sufficiently weighty to override the concerns related to the minor’s right to informational privacy? However, a less antagonistic way of framing this debate is that where the health or reproductive interests of the parents are concerned, serving those interests is also in the interest of the child who depends on the ability of its parents to provide for its daily care.

The suggestion is that, in the near future, genome sequencing may become a standard procedure in prenatal diagnostics and neonatal screening for serious and actionable congenital diseases which lends further urgency to this debate [73–75].

## Recommendations of ESHG: opportunistic screening in genomic medicine

According to earlier ESHG Recommendations on NGS in health care [1], genomic analysis should be as targeted as possible, at least for the time being. Taking account of further developments in science and clinical practice, this new document confirms the 2013 viewpoint that a broader analysis than that needed to answer the diagnostic question raises complex issues in clinical practice. This is not to say that all forms of OGS are a priori unsound. However, if OGS is being offered, it should take the form of pilots combined with rigorous evaluation studies aimed at reducing present uncertainties that could stand in the way of determining its proportionality as a health care service.

(1) Performing a broader analysis than needed to answer the diagnostic question amounts to a form of screening, for which the general framework of screening criteria is applicable. In addressing the question whether such OGS would meet the relevant and widely endorsed criteria for (genetic) screening, ethical principles of proportionality, respect for autonomy, and justice should be considered.

(2) In light of the non-indicated nature of OGS, there is a strong burden of proof that such screening is on balance beneficial for those to whom it is offered. Although so far no evidence of psychosocial harm has emerged, more research is needed. In the light of this, it is too early to recommend OGS as part of the professional standard.

(3) In view of the many uncertainties, directly impacting the required proportionality of any OGS, the ESHG continues to recommend a generally cautious approach. Any OGS should be embedded in adequate pilot and evaluation studies in order to enable optimal decision making about the proportionality of OGS. Priority should be given to well-known, highly penetrant variants, predisposing for genetic disorders which can be adequately and effectively prevented and/or treated. The selection may well be contextual, taking account of both the penetrance of particular variants in a given population, which may differ between populations in Europe, and the capacity of the different health-care systems to integrate relevant, complex, counseling, and (preventive) treatment services for proven bearers of these variants. Apart from genetic and medical uncertainties, and implementation issues, the psychological impact of OGS merits attention. Crucial questions include how to enable patient empowerment and address counseling needs.

(4) Clear procedures and criteria are needed for decision making about the composition and extension of the list of genetic variants included in any OGS, and its implementation. A wider debate, involving all relevant stakeholders, especially patients, is of utmost importance. Patients should not be reduced to the object of well-intended medical deliberations and interventions.

(5) Informed consent should be a central ethical norm in the framework regarding genetic screening generally and OGS particularly. Alternatives such as opting out and, particularly, a coercive offer of OGS are problematic. A multi-step (‘dynamic’) consent approach may be helpful but needs further empirical study. The patient’s right not to know should be respected as far as reasonably possible, while allowing professionals to still inform the patient about specific findings of great importance for the patient’s own health or that of his or her close relatives.

(6) When counseling for OGS, the provisional nature of current knowledge on penetrance should be addressed as well as potential crossovers with research and options for recontacting in case new scientific evidence of clinical relevance arises.

(7) Depending on developing evidence on penetrance and actionability, but also taking account of the resources available for health care in European countries, OGS pilots may be justified to generate data for a future, informed, comparative analysis of OGS and its main alternatives, namely (the offer of) universal genomic screening for highly penetrant, actionable variants, and (more systematic) cascade testing in relatives of probands affected with (avoidable) diseases caused by highly penetrant genetic variants.

(8) OGS in minors for variants leading to later-onset actionable conditions needs further ethical scrutiny. There seem to be no valid principled objections to OGS in children for PGx variants and variants leading to early-onset actionable conditions. Likewise OGS for late-onset disorders could be offered for minors who, because of, for example, profound intellectual disability, are not expected to become competent later (if such targeted OGS would meet the principles of proportionality and justice).
